# Perceived chronic stress influences the effect of acute stress on cognitive flexibility

**DOI:** 10.1038/s41598-021-03101-5

**Published:** 2021-12-08

**Authors:** Katherine Knauft, Alexander Waldron, Mishali Mathur, Vrinda Kalia

**Affiliations:** 1 Department of Psychology, Miami University, 90 N Patterson Ave., Oxford, OH USA; 2 Columbia University Mailman School of Public Health, 722 W 168th St., New York, NY USA

**Keywords:** Human behaviour, Cognitive neuroscience, Stress and resilience

## Abstract

Executive functions are cognitive processes that facilitate goal-directed behavior by enabling us to direct and control our thoughts. Cognitive flexibility is an executive function characterized by the ability to mentally shift between rules, strategies, or tasks. Several studies have reported that acute (brief) stress impairs cognitive flexibility. Even though an individual’s perception of their chronic stress levels is shown to influence effects of future stressors, the interactive effect of acute and perceived chronic stress on cognitive flexibility is not known. We conducted two experiments to address this gap. In both studies, perceived chronic stress was measured using the Perceived Stress Scale. Acute stress was induced using the Cold Pressor Test. Number of perseverative errors on the Wisconsin Card Sorting Test was used as an indicator of cognitive flexibility. In Study 2, we also measured salivary alpha amylase as a marker of the physiological stress response. Data from our two studies are consistent with the hypothesis that an individual’s perception of their chronic stress level may impact the effect of acute stress on perseveration. In Study 1, we observed a significant interaction between acute and perceived chronic stress on perseverative errors, such that only individuals who reported high levels of perceived chronic stress prior to acute stress exposure showed no change in perseveration following the acute stress manipulation. This effect did not differ based on participant sex. In Study 2, we found a similar interaction effect of acute and perceived chronic stress on perseverative errors in an all-woman sample. After identifying salivary alpha amylase responders and non-responders, we observed a strong, negative correlation between perceived chronic stress and perseverative errors amongst the responders only. Our data highlight the value in studying salivary alpha amylase in response to acute stress exposure. Additionally, perceived chronic stress emerged as a key variable in the relationship between acute stress and cognitive flexibility. Overall, our work suggests that future research interested in interrogating moderators in the relationship between acute stress and cognition would benefit from inclusion of measures of chronic stress.

## Introduction

Being flexible enables adaptation to changing environments. However, stress can influence an individual’s flexibility. One aspect of flexibility is cognitive flexibility, which is the ability to shift between sets of rules, strategies, and tasks^1^. During experiences of acute (brief) stress, resources shift away from brain regions implicated in top-down, goal-directed behavior, such as the dorsolateral prefrontal cortex (dlPFC), and towards regions that promote bottom-up threat detection, such as the amygdala, the dorsal anterior cingulate cortex, and the hypothalamus^2^. Cognitive flexibility is a top-down process influenced by stress^3,4^. Acute stress exposure can increase the propensity to make perseverative errors—the repeated use of a strategy or rule that is no longer helpful or effective^3,4^; making these errors is often used as an indicator of a lack of cognitive flexibility^5^.

Several facets of cognitive flexibility have been studied under acute stress conditions^3,4,6–8^. Shields et al.^4^ found increased perseveration in set-shifting following acute stress, particularly for men. The researchers posited that sex differences in the mu-opioid receptor, noradrenergic activity, or dopaminergic activity might explain the stronger stress effects in males. They were unable to test these mechanisms in their sample, but their analyses suggested that cortisol response to acute stress did not account for the inflexibility demonstrated by men. Prior to Shields et al., work by Kudielka and Kirschbaum^9^ noted sex differences in the hypothalamic–pituitary–adrenal axis stress response. Building on this research, Kalia et al.^3^ examined the effects of acute stress on change in perseveration on the Wisconsin Card Sort Test^10^, before and after stress exposure. To investigate prefrontal cortex (PFC) function during acute stress exposure, Kalia et al. measured functional changes in blood oxygenation levels in the prefrontal cortex (PFC) using functional near-infrared spectroscopy (fNIRS). Consistent with the results of Shields et al., Kalia et al. observed that acute stress increased perseveration in males. Moreover, by examining blood oxygen levels in the PFC during acute stress exposure, Kalia et al. were able to show that change in perseverative errors was positively correlated with changes in levels of oxygen in the left dlPFC during recovery. In effect, male participants with larger increases in blood oxygenation in the left dlPFC during recovery tended to have fewer perseverative errors following stress. Overall, these findings suggest that acute stress can lead to perseveration in men and levels of blood oxygenation in the dlPFC may be implicated in this effect.

Perseveration is an aspect of cognitive flexibility that involves the use of a previous mental set or framework that is no longer helpful. Tendencies to perseverate change across the lifespan. Generally, perseveration decreases across age as children gain the ability to construct higher-order rules^11^. Perseveration has previously been characterized as the dissociation between an action and its goal or intent^12,13^. Perseveration has also been positively correlated with psychopathology, such as rumination^14,15^, suicidal behavior^16^, and neurodegenerative diseases^17^. Although Kalia et al.^3^ and Shields et al.^4^ show that perseveration is increased by acute stress exposure, there are also instances when there is no change observed in cognitive flexibility under acute stress conditions^18^ or aspects of cognitive flexibility improve^7^. These findings signal the need for further work on the effect of acute stress on cognitive flexibility, especially work that incorporates individual differences that may be driving specific aspects of cognitive flexibility being examined.

Research on the effects of acute stress on cognitive flexibility is more recent, but previous work has demonstrated similar effects of chronic stress (i.e., prolonged and long-term feeling of stress) on cognitive flexibility^19^. In a thoughtfully designed study, Liston et al. investigated the effect of chronic stress on cognitive flexibility in pre-med students. Liston et al. used the Perceived Stress Scale^20^ to measure levels of chronic stress and found that high levels of perceived chronic stress were associated with reduced functional connectivity of the dlPFC with other areas implicated in attention, including the anterior cingulate and ventrolateral prefrontal cortex. Additionally, participants under high levels of perceived chronic stress exhibited impaired set-shifting, an aspect of cognitive flexibility. Consistent with arguments by McEwen^21^, participants’ perceptions of chronic stress correlated with the magnitude of their set-shifting impairment.

Considering that the dlPFC is implicated in the relationship between cognitive flexibility and both acute^3^ and perceived chronic stress^19^, it is plausible to expect that perceptions of chronic stress may play a role in the relationship between acute stress and cognitive flexibility. Epel et al.^22^ have argued that perceived chronic stress is a key piece of a person’s context that influences how an individual experiences acute stress. In essence, the authors argue that acute stressors are experienced through the lens of contextual factors, including perceived chronic stress. Framed within this perspective, perceived chronic stress could modulate the experience and effects of acute stress. Therefore, we conducted two studies to investigate the interactive effect of acute and perceived chronic stress on cognitive flexibility. It is important to note that Liston et al.^19^ did not look at perseveration, thus the effect of perceived chronic stress on perseveration remains unknown. Our secondary goal was to address this gap.

## Study 1

In this study, we used the experimental design outlined in Kalia et al.^3^ with the addition of perceived chronic stress as a variable. Participants were assigned to either the experimental condition, in which they were exposed to a physiological stressor, or the control condition, in which the physiological stimulus was presented without the stressful element (i.e., cold temperature). Based on findings from Kalia et al., we hypothesized that exposure to acute stress would influence perseveration. Specifically, we expect that participants in the acute stress condition will show greater perseveration in the set-shifting task following acute stress exposure than those in a control condition. Second, also consistent with Kalia et al., we hypothesize that we will observe sex differences in the effects of acute stress on cognitive flexibility. We expect the negative effect of acute stress on perseveration will be stronger for male than for female participants.

Perceptions of chronic stress have been found to shift the physiological^23,24^ and affective^25^ response to additional acute stressors. In effect, individuals who perceive that they are under high levels of chronic stress react differently to additional stress compared to individuals reporting lower levels of perceived chronic stress. Thus, perceived chronic stress may be a key moderating factor in the relation between acute stress and cognitive flexibility. Our final hypothesis was that perceived chronic stress and acute stress exposure will interact to influence perseveration. Considering that this effect has not been reported before, we did not develop any specific hypothesis regarding the specific nature of the interaction.

### Study 1 Methods

#### Participants

Participants (N = 95; Female = 54; Range_*Age*_: 18–26; Mean_*Age*_ = 19.24) were recruited using the Miami University psychological research pool of undergraduate students in exchange for partial course credit. Sex, age, and race of participants did not differ significantly between conditions (*p*s > 0.476). The majority of participants identified as White (76.8%). The remaining participants identified as Asian (14.7%), Hispanic (4.2%), African-American or Black (3.2%), or preferred not to respond (1.1%). All methods were carried out in accordance with relevant guidelines and regulations. All study procedures were approved by the Miami University Department of Psychology Departmental Review Board; the protocol was carried out in accordance with recommendations from the Institutional Review Board. Informed consent was obtained from each participant prior to participation.

#### Materials and measures

##### Perseveration: Wisconsin Card Sorting Test (WCST)^10^

Participants’ perseveration was assessed using a computerized version of the WCST^10^. The WCST asks participants to sort cards according to one of three possible dimensions^26^. At any given time, one dimension is correct, and participants must identify the correct sorting dimension through trial and error. Thus, the WCST is thought to measure aspects of cognitive flexibility linked to set-shifting^27^. Participants are given a deck of cards to be sorted into one of four piles. Participants are not told which sorting rule to apply; instead, they must guess which dimension to sort by based on feedback on if their choice was correct^28^. After a participant successfully sorts a series of six cards, the correct sorting dimension changes and participants must shift their sorting strategy to identify the new correct dimension.

Though the WCST provides several measures of performance, the primary outcome of interest within the present study is perseverative errors, or the number of times participants continue to sort by a previous, now incorrect dimension, despite receiving feedback that they made an error. Perseverative errors is one of the most common outcome variables used to evaluate cognitive flexibility in the WCST^29^. Miyake et al.^27^ suggest that perseverative errors on the WCST primarily measure set-shifting, rather than other executive functions. Previous work in studies of individuals with head injuries finds that perseverative errors on the WCST are significantly related to metabolism in the dlPFC^30^. Finally, Kalia et al. found perseverative errors was the WCST outcome most impacted by acute stress^3^, consistent with previous literature showing perseverative errors indicate a lack of cognitive flexibility^31–33^.

##### Perceived Chronic Stress: Perceived Stress Scale (PSS)^20^

The Perceived Stress Scale is a ten-item self-report measure that assesses an individual’s experience of how overwhelming and uncontrollable their lives have generally felt over the last month (e.g., *In the last month, how often have you found that you could not cope with all the things you had to do?*). Responses were measured on a five-point Likert scale ranging from 0 (“*Never*”) to 4 (“*Very often*”). Items were reverse scored as indicated in the original scale and summed to create a total score representing perceived stress. Possible scores range from 0 to 40. Within this sample we observed scores ranging from 5 to 31. Higher scores indicate more perceived stress (α = 0.86).

##### Stress induction: traditional cold pressor test (CPT)^33,34^

Participants who were randomly assigned to the stress condition (n = 44) were asked to place their non-dominant hand in ice-cold (1–3 °C) water for 3 min, while participants who were randomly assigned to the control condition (n = 51) were asked to do the same in room-temperature water (35–37 °C) water. The experimenters monitored the participants during the task and kept a neutral demeanor at all times, thus ensuring that they did not provide participants with any verbal or facial indicators of reassurance.

##### Manipulation check: subjective stress measure

Participants were asked to complete a single-item subjective stress assessment before and after the acute stress manipulation. The first (pre-manipulation measurement) took place immediately prior to the first administration of the WCST. The second assessment of subjective stress took place immediately after the completion of the acute stress or control manipulation. Both times, participants were asked to rate their current stress level on a scale ranging from 0 to 10. In our sample, scores ranged from 0 to 8.

#### Procedure

**Figure 1:**
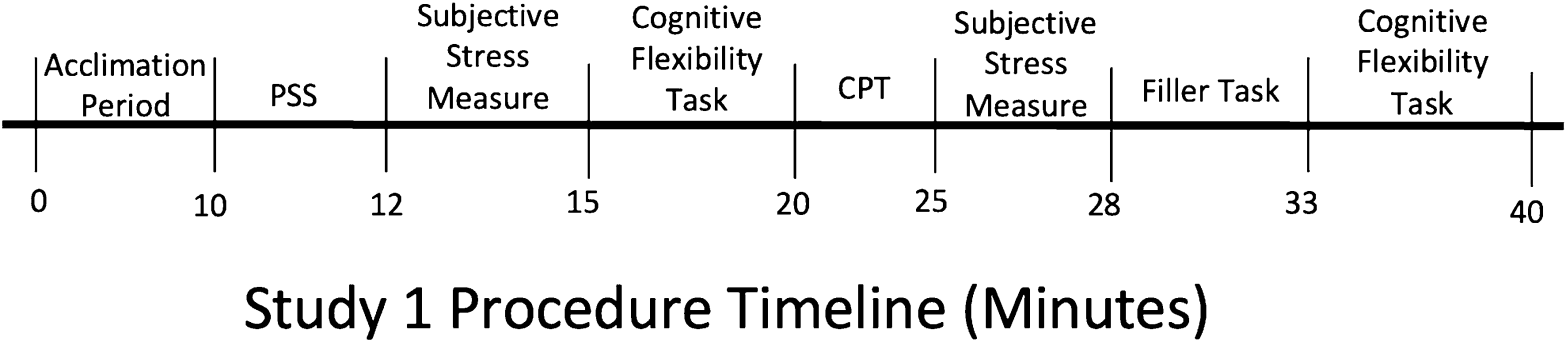
Schematic of study 1 procedure; *PSS* perceived chronic stress scale, *CPT* cold pressor task.

All data were collected in the afternoon. Participants arrived at the lab individually and upon arrival were randomly assigned to the acute stress or control condition. The randomization was determined prior to the start of the study with the creation of an excel sheet in which ID numbers were randomly assigned to one of the two conditions. These randomly distributed ID numbers were consequently used to assign participants to the control or experimental condition. After signing a consent form, the participants completed the Perceived Stress Scale. After this, participants completed the WCST before beginning the acute stress induction (via CPT) or the non-stress control task. Immediately after exposure to the acute stress or control manipulation, participants rated their subjective stress level. Participants were then given a 10-min filler task before taking the WCST a second time. Once the participants were finished with the WCST, they were debriefed and exited the study. See Fig. 1 for a schematic of the study procedure. See Table 1 for demographic information and Table 2 for descriptive statistics.

**Table 1:** Demographic information for participants in Study 1 and Study 2 in percentages.

Condition	Sex		Race/ethnicity	
**Study 1**				
Control	Male	39.2	African-American/Black	3.9
			Asian	9.8
	Female	60.8	White	82.4
			Hispanic	3.9
Experimental	Male	45.5	African American/Black	2.3
			White	70.5
	Female	52.3	Asian	20.5
			Hispanic	4.5
			Prefer not to respond	2.3
	χ^2^ = 0.51; *p* = 0.476		χ^2^ = 2.47; *p* = 0.481	
**Study 2**				
Control	Male	0	African-American	6.3
			Asian or Asian American	12.5
	Female	100	White	62.5
			White and Asian	12.5
			Hispanic	6.3
Experimental	Male	0	African and Hispanic	5.9
	Female	100	White	88.2
			White and Asian	5.9
	N/A		χ^2^ = 6.31; *p* = 0.277	

chi-square and p values indicate the magnitude of the differences in frequencies between the acute stress and control condition for each variable.

**Table 2:** Descriptive statistics.

Variables	*Acute stress condition*			*Control condition*			*t*	*p*	*Total*		
	n	Mean	SD	n	Mean	SD			N	Mean	SD
**Study 1**											
PSS	44	15.30	6.65	51	18.00	5.90	2.10	0.038*	95	16.75	6.37
Female participants	23	16.65	7.27	31	18.16	5.29	0.88	0.381	54	17.52	6.19
Male participants	20	13.80	5.85	20	17.75	6.88	1.96	0.058	40	15.78	6.62
Subjective stress (Pre)	43	2.96	2.20	51	3.53	2.05	1.28	0.203	94	3.27	2.13
Female participants	23	3.13	2.36	31	3.52	1.81	0.68	0.499	54	3.35	2.05
Male participants	20	2.78	2.03	20	3.55	2.44	1.09	0.285	40	3.17	2.25
Subjective stress (Post)	44	3.83	1.80	51	3.07	1.94	− 1.98	0.051	95	3.42	1.90
Female participants	23	4.17	1.83	31	3.21	1.90	− 1.88	0.066	54	3.62	1.91
Male participants	20	3.43	1.77	20	2.85	2.03	− 0.96	0.343	40	3.14	1.91
Perseverative errors (Pre)	44	4.00	1.71	51	4.39	2.12	0.98	0.321	95	4.21	1.90
Female participants	23	3.91	1.83	31	4.42	1.89	0.99	0.329	54	4.20	1.87
Male participants	20	4.20	1.58	20	4.36	2.48	0.23	0.821	40	4.28	2.05
Perseverative errors (Post)	44	3.36	2.07	51	3.45	2.14	0.20	0.841	95	3.41	2.10
Female participants	23	3.35	1.82	31	3.71	2.04	0.67	0.503	54	3.56	1.94
Male participants	20	3.55	2.28	20	3.05	2.28	− 0.69	0.493	40	3.30	2.27
**Study 2 (all women)**											
PSS	17	20.24	5.62	16	18.94	6.55	− 0.61	0.545	33	19.61	6.03
Subjective stress	17	4.59	1.80	16	3.31	1.70	− 2.09	0.045*	33	3.97	1.85
Subjective discomfort	17	8.18	1.33	16	2.44	1.50	− 11.61	< 0.001***	33	5.39	3.23
Subjective difficulty	17	7.24	1.68	16	2.56	1.55	− 8.30	< 0.001***	33	4.97	2.86
Perseverative errors	17	3.65	1.68	16	3.25	2.02	− 0.57	0.57	33	3.45	1.99

t- and p values indicate the magnitude of the differences between the acute stress and control condition for each variable; *p < 0.05; ***p < 0.001 measures of subjective stress, discomfort, and difficulty in Study 2 occurred following the acute stress or control manipulation.

#### Data analytic plan

Analyses were conducted using SPSS version 26. To test whether the experimental manipulation induced acute stress in participants, we used a mixed-model ANOVA to test the effect of condition on subjective ratings of stress from before the acute stress manipulation to after the manipulation. Because we are interested in understanding how perceived chronic stress interacts with acute stress effects, we also conducted bivariate correlations between perceived chronic stress and pre- and post-manipulation subjective stress measures. These correlations were conducted separately for the acute stress and control conditions.

To test our primary hypothesis that acute and chronic stress interact to influence cognitive flexibility, we conducted a mixed-model ANOVA predicting perseverative errors on the WCST pre- and post-manipulation. Condition was added as a between-subjects variable. In order to ensure that our test for possible interactions between acute and perceived chronic stress was interpretable^35^, participants were grouped according to high, medium, and low perceived chronic stress using their score on the PSS. Splitting participants into groups according to high, medium, and low perceived chronic stress was done to be consistent with the use of high and low chronic stress groups reported by Liston et al.^19^. In our study, participants were considered high on perceived chronic stress if their perceived chronic stress rating was one standard deviation or more above the mean. Participants were considered low on perceived chronic stress if their perceived chronic stress rating was one standard deviation or more below the mean. All participants with scores within one standard deviation of the mean were considered to have medium levels of perceived chronic stress. This variable was added to the model as a second between-subjects variable.

### Study 1 Results

#### Acute stress manipulation check: subjective reports

Ratings of subjective stress were compared between the acute stress and control conditions using a mixed-model ANOVA, wherein time (subjective ratings pre- and post-manipulation) was included as a within-subjects measure and condition (acute stress and control) was included as a between-subjects measure. A significant time by condition interaction emerged, *F*(1, 92) = 17.58, *p* < 0.001, η^2^ = 0.16. The individuals in the control condition reported a decrease in subjective stress from pre-manipulation (M = 3.53) to post-manipulation (M = 3.07), whereas individuals in the stress condition showed an increase in subjective stress from pre-manipulation (M = 2.97) to post-manipulation (M = 3.83). Thus, our stress manipulation was successful.

Because perceived chronic stress was a key variable in our analyses and was assessed prior to the stress manipulation, we conducted bivariate correlations to ensure that the association between perceived stress and subjective stress did not differ between the stress and control conditions. The analysis revealed that the association between perceived chronic stress and subjective acute stress was nearly identical for the two groups: stress (pre-manipulation: *r* = 0.57; *p* < 0.001, post-manipulation: *r* = 0.55; *p* < 0.001) and control (pre-manipulation: *r* = 0.56; *p* < 0.001, post-manipulation: *r* = 0.56; *p* < 0.001). Thus, our data suggest that subjective ratings of stress were strongly associated with perceived chronic stress regardless of the condition to which participants were assigned.

#### Effects of acute and perceived chronic stress on cognitive flexibility

To test our primary hypothesis that perceived chronic stress will interact with acute stress exposure to impact perseverative errors, we ran a mixed-model ANOVA. Time (Perseverative errors pre- and post-manipulation) was included as a within-subject factor. Condition (acute stress vs. control) was added as a between-subjects factor along with sex, as the effect of acute stress on perseverative errors has been found to differ on the basis of sex^3,4^. Finally, levels of perceived chronic stress (low vs. medium vs. high) was included as a between-subjects factor.

**Figure 2:**
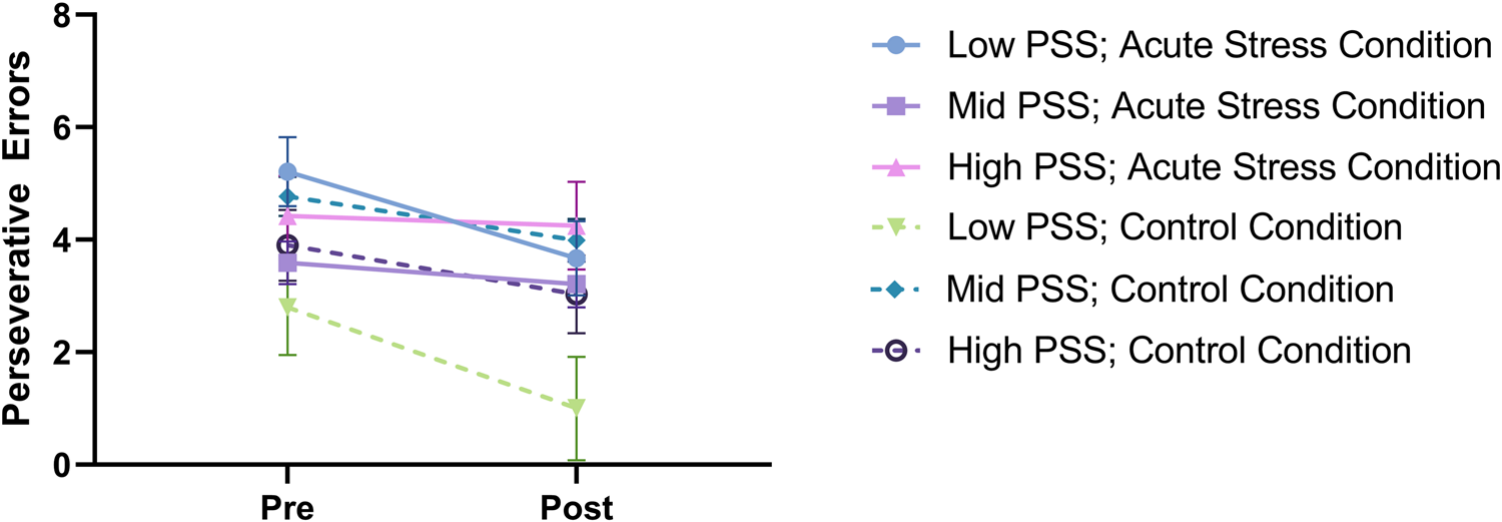
Number of perseverative errors for high, medium, and low perceived chronic stress groups across the acute stress and control conditions; error bars = standard error; dashed line = control condition; figure includes all participants (n_control_ = 51, n_stress_ = 44).

A significant within-subject effect of time emerged, *F*(1, 83) = 10.77, *p* = 0.002, η^2^ = 0.12. Across groups, participants committed fewer perseverative errors (M = 3.45) after the manipulation than they did prior to the manipulation (M = 4.23). None of the between-subject factors interacted significantly with time (*p*s > 0.312). Similarly, no between-subject effects of condition or sex emerged as significant (*p*s > 0.244). A significant interaction between condition and perceived stress level did emerge, *F*(2, 83) = 4.38, *p* = 0.016, η^2^ = 0.10, suggesting that the effects of condition on perseverative errors differed based on the participant’s reported experience of perceived chronic stress (see Fig. 2).

Post-hoc tests reveal that only individuals with low, *F*(1, 13) = 13.37, *p* = 0.003, and medium levels of perceived chronic stress, *F*(1, 62) = 13.37, *p* = 0.009, show a main-effect of time. For individuals experiencing low and medium levels of perceived chronic stress, the number of perseverative errors decreased from Time 1 to Time 2. Individuals experiencing high levels of perceived chronic stress did not show a difference in perseverative errors from Time 1 to Time 2, *F*(1, 14) = 0.71, *p* = 0.412.

Similarly, post-hoc tests reveal that individuals experiencing low perceived chronic stress, *F*(1, 13) = 8.33, *p* = 0.013, and medium perceived chronic stress, *F*(1, 62) = 6.10, *p* = 0.016, showed a significant difference between the control condition and the stress condition. In contrast, individuals experiencing high perceived chronic stress did not, *F*(1, 14) = 0.82, *p* = 0.380. In effect, only individuals experiencing low and medium levels of perceived chronic stress showed less cognitive flexibility in the stress condition compared to the control condition.

## Study 2

For Study 2, we altered the procedure used in Study 1 in three ways. First, we recruited an all-women sample. This was done because sex and gender effects in studies of acute stress on cognitive flexibility have been mixed; some have observed differences^3,4^ whereas others have not^6,8,36^. Since sex differences were not found in Study 1 and women are more likely to develop stress-related disorders^37,38^, we chose to focus on women for Study 2.

Second, we measured salivary alpha amylase in response to acute stress exposure because it is an indicator of sympathetic response, which has been implicated in the impact of acute stress on cognition^6,39,40^. Additionally, the sympathetic response to acute stressors may be influenced by perceived chronic stress^23,24^. To clarify the role of the sympathetic response in the interactive effect of stress on perseveration, we will measure salivary alpha amylase (sAA), a marker of sympathetic nervous system activity^41^.

Third, we increased the time between the measurement of chronic stress and acute stress exposure. In Study 1, we found that perceived chronic stress correlated with current subjective stress pre- and post- acute stress manipulation. Correlations between chronic stress and current subjective stress following an acute stressor have been previously reported^42^. Given shared variance between perceived chronic stress and current subjective stress, we aimed to ensure that our findings are being driven by perceived chronic stress. To do so, we conducted a two time point study. Specifically, we measured perceived chronic stress 2 weeks prior to the acute stress manipulation thereby eliminating any possible confounding of the experience of perceived chronic stress and current subjective stress.

We have three primary hypotheses for Study 2. First, we expect to find that individuals in the acute stress condition exhibit more perseverative errors on the WCST than those in the control condition^3,4^. Second, we expect that participants in the acute stress condition will show increases in salivary alpha amylase following the manipulation, whereas individuals in the control condition will not^43^. Third, consistent with findings of Study 1, we expect to find that the impact of acute stress exposure on perseverative errors will vary as a function of individual differences in the experience of perceived chronic stress.

### Study 2 Methods

#### Participants

**Figure 3:**
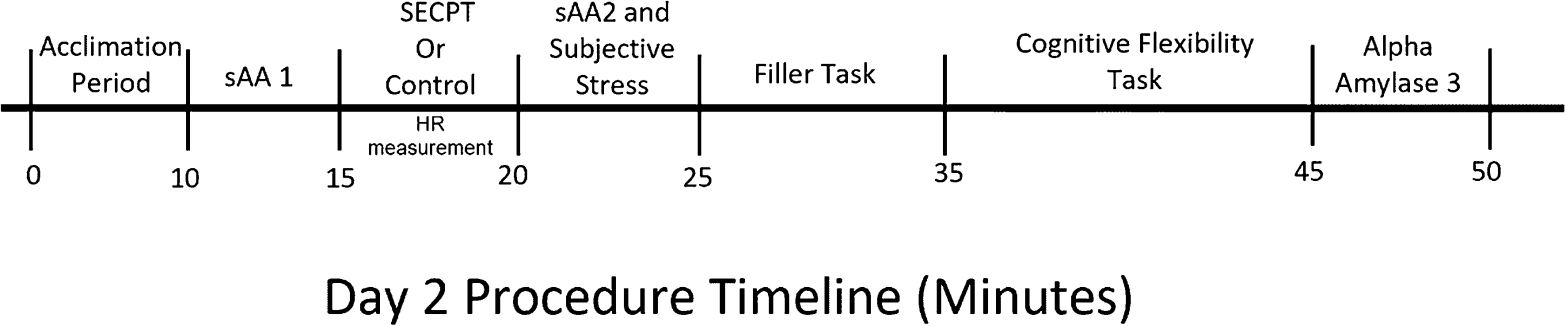
Schematic of experimental procedure timeline for Day 2; *sAA* salivary alpha amylase, *SECPT* socially evaluated cold pressor test.

Women (N = 47) were recruited from the Miami University subject pool to complete a 2-day study. On the first day, participants completed demographics and an assessment of their perceived chronic stress levels. On the second day, participants were randomly assigned to either acute stress condition or the control condition before completing the WCST (see Fig. 3).

It is relevant to note that due to the unexpectedness of the university’s shutdown in response to the COVID-19 pandemic, we were unable to complete Day 2 for 14 of these participants. Thus, the final sample consists of 33 participants (Range_*Age*_ = 18 – 20; Mean_*Age*_ = 18.7) who have provided data for both time points. Age did not differ significantly across conditions, *t*(31) = 0.70, *p* = 0.490. The majority of the final sample (75.8%) reported their race as white with remaining participants identifying as Asian or Asian-American (6.1%), African-American (3.0%), or multi-racial (15.1%). All methods were carried out in accordance with relevant guidelines and regulations. All study procedures were approved by the Miami University Department of Psychology Departmental Review Board; the protocol was carried out in accordance with recommendations from the Institutional Review Board. Informed consent was obtained from each participant prior to participation.

#### Materials and measures

##### Perceived Chronic Stress: Perceived Stress Scale (PSS) ^20^

As in Study 1, perceived chronic stress was assessed via the Perceived Stress Scale (α = 0.86). Within the present sample, scores ranged from 5 to 31.

##### Stress induction: socially-evaluated cold pressor test for groups^44^

The socially-evaluated cold pressor test for groups is an adaptation of the socially-evaluated cold pressor test^45^, combining aspects of the traditional cold pressor test^33,34^ with social evaluative components and allowing for group administration of the stress task^44,45^.

Participants were exposed to the experimental manipulation in groups of four. In the stress condition participants placed their non-dominant hand in ice-cold (1–3 °C) water for 3 min, whereas in the control condition were asked to do the same in room-temperature (35–37 °C) water. Prior to placing their hand in the water, participants were told that cameras set up around the room were recording them and would be coded for changes in their facial expressions and emotions. The cameras in the room were not on and no recording of the session took place. Additionally, the experimenters kept a neutral demeanor at all times thus ensuring that they did not provide participants with verbal or facial indicators of reassurance.

##### Manipulation check: subjective stress measure

To assess subjective stress following stress induction, participants completed three items: one assessing how hard the stress task was, one assessing how uncomfortable the stress task was, and one assessing how stressed they were currently. All three items were assessed on a ten-item Likert scale from “not at all” (1) to “very much” (10). For the items assessing how uncomfortable the task was and how hard the task was, scores ranged from one to 10. For the item assessing how stressed participants were, scores ranged from one to eight.

##### Physiological marker of acute stress: salivary alpha amylase

Participants provided three saliva samples on Day 2 (see Fig. 3). These saliva samples were used to measure salivary alpha amylase (sAA), which we are treating as a proxy for current sympathetic nervous system activity^41^. Saliva samples were processed using dry chemistry (Nipro Corp., Japan), with a handheld monitor and a disposable test strip which participants place under their tongue for 10 s. The dry chemistry method has been used previously to evaluate sympathetic nervous system activity following acute stress^46,47^. This method uses a disposable test strip and hand-held monitor to test the concentration of sAA in saliva^48^. The color of the test paper contained in the test strip changes color in proportion to the amount of sAA in the saliva sample. The handheld monitor then measures the intensity of the color change. Shetty et al. found high levels of accuracy, precision, and consistency of sAA measurement using this approach^48^. In particular, reports suggest close agreement between sAA levels measured via the dry chemistry biosensor and other methods of analysis—including a laboratory-based automated clinical chemistry analyzer^48^ and conventional assays, so long as saliva collection methods are consistent^49^—indicating convergent validity between sAA measurement techniques. See Fig. 3.

##### Perseveration: Wisconsin Card Sorting Test (WCST)^10^

Like Study 1, cognitive flexibility in Study 2 was measured via perseverative errors on the Wisconsin Card Sorting Test.

#### Procedure

Participants were invited to the lab twice, with the visits taking place approximately 2 weeks apart. Prior to their second visit, participants were asked to refrain from eating or drinking beverages other than water an hour before their lab visit, to reduce any influence of food intake on sAA measurement^50^. The second lab visit was scheduled for the afternoons, to control for the effect of the diurnal course sAA as recommended^51^. Participants were randomly assigned to the stress or control condition using procedures described in Study 1.

On day 1, demographics and baseline measurements of perceived chronic stress using the Perceived Stress Scale^20^ were taken. On day 2 (which ranged from 11 to 19 days after the first day; *M*_days_ = 13.61), participants completed the acute stress induction or control task. Upon arrival to the lab, participants acclimated to the lab environment for ten minutes. During this time baseline assessment of their current levels of stress were made and at the end of this acclimatization the first salivary sample was taken. Then, participants were exposed to the stress manipulation. Immediately following stress induction, the second salivary sample was taken, after which participants completed the subjective stress measure. After a filler task, the WCST was administered to assess changes in cognitive flexibility following acute stress. Once participants completed the WCST, a final saliva sample was taken.

#### Data analytic plan

Analyses were conducted using SPSS version 26. To address our first hypothesis, differences in subjective acute stress as a function of group (Stress vs. Control) were assessed. Because our hypothesis is that individuals in the acute stress conditions will rate the task to be more subjectively stressful, we used one-tailed independent samples t-tests to compare the ratings of the three subjective measures between the acute stress and control conditions.

We used mixed-model ANOVAs to test our second hypothesis: whether salivary alpha amylase, a physiological indicator of acute stress, differs between the acute stress and control conditions across time. As we are interested in understanding the relationships between indices of the physiological acute stress response, perceived chronic stress, and cognitive flexibility, we correlated levels of salivary alpha amylase with perceived chronic stress and perseverative errors for the control and acute stress conditions separately. As there is evidence that individual variability in sAA response influences performance during stress^52^, we used the procedure outlined by Becker and Rohleder^52^ to identify sAA responders and non-responders. We conducted correlations between the relevant variables for these two groups as well. Because we expect salivary alpha amylase to show levels of skew and kurtosis greater than |1|, non-parametric Spearman’s Rho correlations were used for all correlational analyses.

To address our final hypothesis, that the effect of acute stress on cognitive flexibility was qualified by an interaction between acute stress and perceived chronic stress, we used regression-based moderation analyses, with acute stress as a predictor, perceived chronic stress as a moderator, and perseverative errors on the WCST as the dependent variable.

### Study 2 Results

#### Acute stress manipulation check: subjective reports

**Figure 4:**
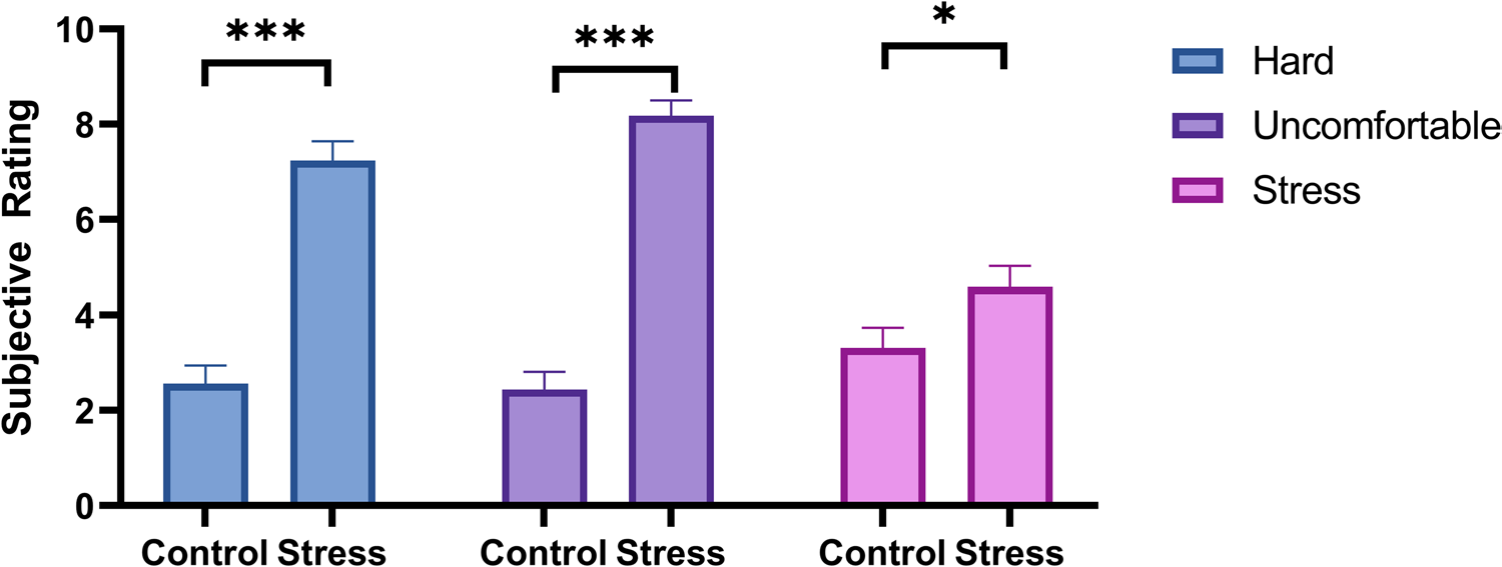
Comparison of subjective ratings following the CPT between conditions; error bars = standard error; *p < 0.05; ***p < 0.001; figure includes all participants (n_control_ = 16, n_stress_ = 17).

Compared to those in the control condition, participants in the stress group rated the CPT significantly more difficult (stress: M = 7.24, SD = 1.68, control: M = 2.56, SD = 1.54), *t*(31) = − 8.30, *p* < 0.001, *d* = 2.90, more uncomfortable (stress: M = 8.18, SD = 1.33; control: M = 2.44, SD = 1.50), *t*(31) = − 11.61, *p* < 0.001, *d* = 4.05, and more stressful (stress: M = 4.59, SD = 1.80; control: M = 3.31, SD = 1.70), *t*(31) = − 2.09, *p* = 0.023, *d* = 0.7. See Fig. 4. These differences between conditions remained significant when two-tailed independent samples t-tests were used. Thus, our stress manipulation was successful. Importantly, the subjective ratings of stress, task difficulty, and discomfort were not significantly correlated with perceived chronic stress when conditions were analyzed either separately or together (*p*s > 0.148).

#### Physiological marker of acute stress response: alpha amylase

**Figure 5:**
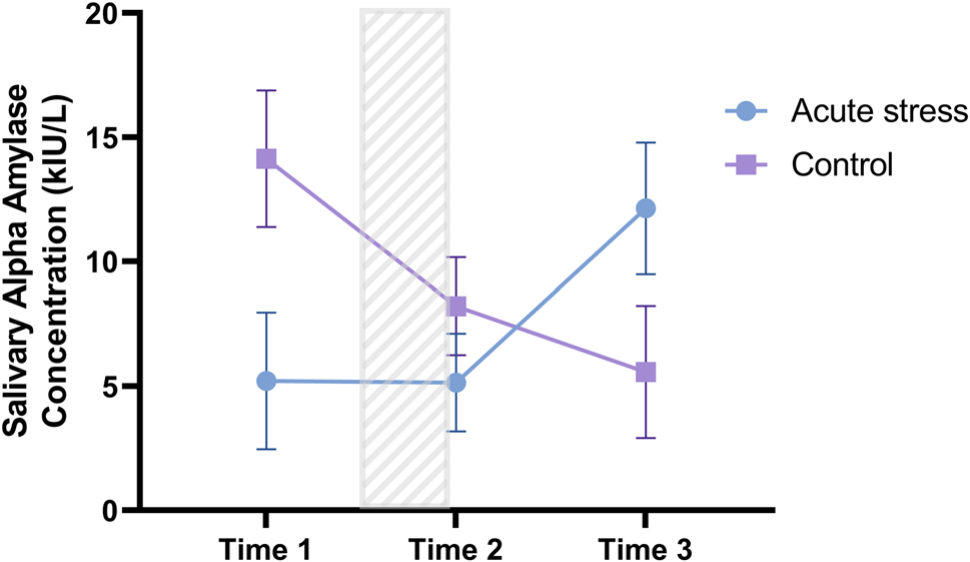
Comparisons between conditions of salivary amylase at baseline, immediately after the CPT, and after completion of the Wisconsin Card Sorting task; figure includes all participants (n_control_ = 16, n_stress_ = 17); error bars = standard error.

To examine differences in salivary alpha amylase levels between the stress and control groups at each time-point, a repeated measures ANOVA with log-transformed sAA data was conducted. Time was added as a within-subjects factor and condition (stress vs. control) as a between-subjects factor. Main effects of time, *F*(2, 54) = 0.35, *p* = 0.704, η^2^ = 0.01, and condition, *F*(1, 27) = 0.17, *p* = 0.686, η^2^ = 0.01, were not significant. There was, however, a significant interaction between time and condition, *F*(2, 54) = 4.10, *p* = 0.022, η^2^ = 0.13, suggesting that individuals in the stress and control conditions showed different patterns of change in sAA responses (see Fig. 5). Planned follow-up independent samples t-tests (two-tailed) showed that there was no significant difference between the stress and control groups sAA responses at Time 1, *t*(27) = 1.89, *p* = 0.067, Time 2, *t*(28) = 0.97, *p* = 0.34, or Time 3, *t*(27) = -1.76, *p* = 0.089.

We compared change in sAA response (determined by subtracting levels at Time 3 from levels at Time 1) between stress and control conditions. When change in sAA response is compared between conditions, significant differences emerge between the stress (M = 0.23; SD = 0.57) and control (M = -0.30; SD = 0.58) condition, *t*(26) = -2.44, *p* = 0.022. This suggests that the change in levels of sAA from pre- to post-stress significantly differed between groups. Specifically, the change scores indicate that from Time 1 to Time 3, sAA increased in the stress group, but decreased in the control group. See Fig. 5.

**Figure 6:**
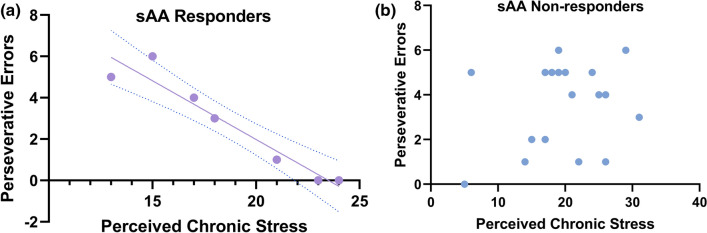
Associations between perceived chronic stress and post-acute stress perseverative errors for sAA responders (**a**; n = 7) and sAA nonresponders (**b**; n = 26); dotted lines indicate 95% confidence interval.

Because levels of sAA differ across individuals based on factors such as dietary habits^50^, it was important to us to not just consider relative change from baseline, but to identify salivary alpha amylase responders versus non-responders. Individuals whose sAA levels increased more than 10 percent and more than 10 kIU/L from pre- to post-stress induction were classified as sAA responders^52^. This analysis identified 7 responders (5 out of the total 17 participants in the stress group and 2 out of the total 16 participants in the control group). Importantly, scores on the perceived chronic stress scale were highly negatively correlated with perseverative errors (*r* = -0.96; *p* < 0.001) in these sAA responders. This correlation was much smaller, positive, and not significant in nonresponders (*r* = 0.329, *p* = 0.135). See Fig. 6. No other associations emerged as significant.

#### Effects of acute and perceived chronic stress on cognitive flexibility

To test our primary hypothesis that previous perceived chronic stress will modulate the effect of acute stress on cognitive flexibility, we ran a moderation analysis investigating the degree to which the effect of Day 2 condition (Acute stress or control) on perseverative errors is moderated by reports of perceived chronic stress measured on Day 1. Analyses were conducted using Hayes’ PROCESS macro^53^.

The overall statistical model did not explain a significant proportion of variance in perseverative errors on the WCST, *R*^2^ = 0.21, *F*(3, 29) = 2.64, *p* = 0.068. This is likely because the small sample (n = 33 who completed Day 2) left the statistical analysis underpowered^54^. In spite of this, acute stress, perceived chronic stress, and the interaction emerged as significant predictors of post-stress perseverative errors (see Table 3). Being in the acute stress condition was associated with an increase in perseverative errors compared to the control group, *B* = 6.05, *t*(29) = 2.68, *p* = 0.012. Similarly, individuals who reported a higher level of perceived chronic stress on Day 1 tended to commit more perseverative errors, *B* = 0.17, *t*(29) = 2.31, *p* = 0.028. However, these results must be interpreted with the significant interaction between condition and perceived chronic stress in mind, *B* = -0.29, *t*(29) = -2.64, *p* = 0.013. The inclusion of the interaction term led to an increase in *R*^2^ of 0.204, *F-change* (2, 29) = 3.76, *p* = 0.035, compared to the model that included only main effects.

**Table 3:** Regression model for Study 2 moderation analysis.

Variable	*B*	*SE*	*T*	*p*
Condition	6.05	2.26	2.68	0.012
Perceived stress	0.17	0.07	2.31	0.023
Interaction	− 0.29	0.11	− 2.64	0.013

B is the unstandardized coefficient.

**Figure 7:**
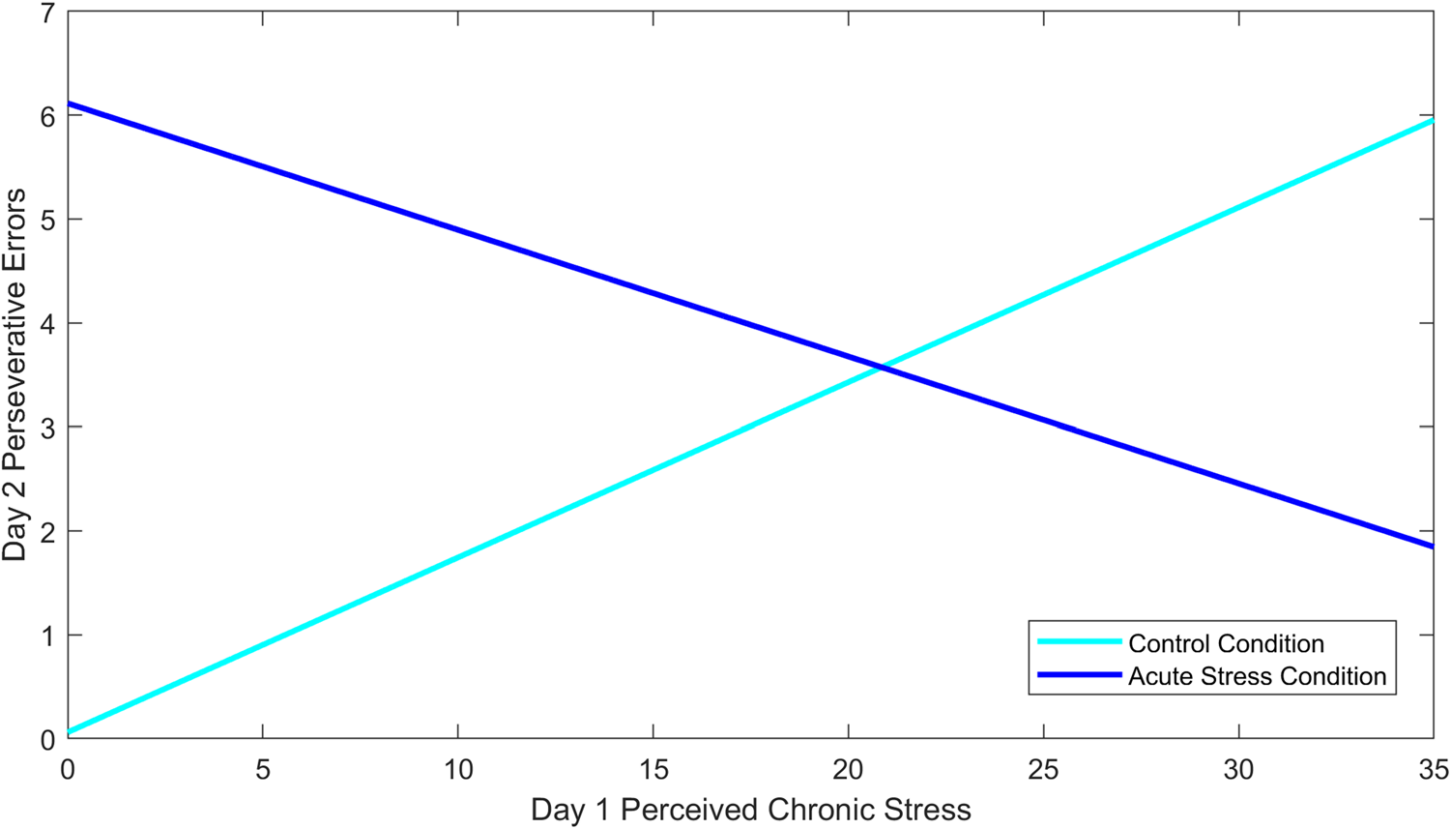
Effect of acute stress condition and day 1 perceived stress score on perseverative errors during the Wisconsin Card Sorting Test; figure includes all participants (n_control_ = 16, n_stress_ = 17).

The Johnson-Neyman technique^55^ indicates that the effect of acute stress on perseverative errors is significant when reported perceived stress is below 15.06. This suggests that women in the acute stress condition who were experiencing low levels of perceived chronic stress (greater than half a standard deviation below the mean; see Fig. 7) exhibited more perseverative errors than their counterparts in the control condition. Women who were experiencing medium levels of perceived chronic stress did not exhibit any effect of acute stress condition. However, women participants who were experiencing very high levels of perceived chronic stress (i.e., scores above 30.20) exhibited higher perseverative errors in the control condition compared to the acute stress condition.

## Discussion

We conducted two studies to examine the degree to which the impact of acute stress on cognitive flexibility varied depending on individuals’ perceived chronic stress. The goals of the studies were twofold. Our primary goal was to understand the interactive relationship between perceived chronic stress and acute stress exposure in cognitive flexibility. Data from both studies supported our primary hypothesis that reported perceived chronic stress would interact with an individual’s exposure to acute stress to influence cognitive flexibility as measured by the WCST.

Our secondary goal was to examine salivary alpha amylase, a marker of acute stress, in relation to cognitive flexibility and perceived chronic stress. In Study 2, we found that salivary alpha amylase increased across time for women in the acute stress condition, but decreased for those in the control condition. Furthermore, we identified sAA responders and non-responders. Within the small number of sAA responders, we observed a strong negative association between perceived chronic stress and perseverative errors. Women who were sAA responders and experiencing high levels of perceived chronic stress tended to have fewer perseverative errors on the WCST. Below we discuss the findings from the two studies in more detail.

### Perceived chronic stress and acute stress interact to impact perseveration

Data from our studies are consistent with the hypothesis that an individual’s perceptions of chronic stress play a role in how they experience acute stressors^19^. In particular, an individual’s perception of chronic stress may interact with acute stress exposure to impact their cognitive flexibility. Within Study 1, we observed a significant interaction between acute stress exposure and perceived chronic stress with implications for cognitive flexibility pre- and post-manipulation (acute stress v. control). In effect, the impact of our manipulation on the number of perseverative errors an individual made depended on whether they reported low, medium, or high levels of perceived chronic stress. To the best of our knowledge, we are the first to report this finding.

Post-hoc tests showed that only individuals who were experiencing low and medium levels of perceived chronic stress exhibited a significant change in the number of perseverative errors due to the manipulation. In contrast, those experiencing high levels of perceived chronic stress did not see any change in flexibility due to condition. As stress-induced plasticity can be considered adaptive in the short-term^19^, our data suggest that individuals with high perceived chronic stress may be less likely to experience adaptive plasticity in the face of acute stress.

These findings contrast work by Goldfarb et al.^7^, who also used the CPT to induce acute stress but did not report perceived chronic stress as a significant predictor of cognitive flexibility under acute stress. This may be due to a couple of reasons. First, cognitive flexibility is a complex construct that can be measured in several ways, and measures of cognitive flexibility may exhibit differential relations with stress^56^. To measure cognitive flexibility, Goldfarb et al. used a task that asked participants to switch between trials of either ignoring or remembering and responding to (i.e., updating) probe figures presented after an initial set of figures. Through this task, Goldfarb et al. assessed both updating and task-switching processes, which are respectively localized to striatal and prefrontal networks. In contrast, our focus was perseveration during set-shifting, which has been localized to the PFC^17^. As our tasks targeted different aspects of cognitive flexibility, the divergence in findings may reflect differences in effects of stress of these aspects of flexibility. Second, Goldfarb et al.^7^ had both males and female participants in their sample (N = 38), which may have influenced their ability to observe an effect. Regardless, the present work provides evidence that the perception of chronic stress may be an important moderating factor when considering cognitive stress effects. These results should provide impetus for greater examination of the interactive effects of acute and chronic stress on cognitive flexibility.

In Study 2, we only studied women and only measured cognitive flexibility post-manipulation. The moderation analyses were underpowered, but observed results are consistent with Study 1. Our data revealed that, when experiencing low levels of perceived chronic stress, women in the acute stress condition had more perseverative errors than women in the control condition. This finding provides evidence for acute stress-induced perseveration, such that the ability to be flexible was impacted by the acute stress exposure. However, women experiencing average to high levels of perceived chronic stress did not show an effect of acute stress exposure. Finally, women experiencing very high levels of perceived stress showed perseveration under control conditions. This pattern of results stands in contrast to Radenbach et al.^57^ who found that acute and chronic stress interactively influenced decision-making in male participants. Interestingly, those reporting high chronic stress showed more rigid and habitual decision-making under acute stress. It is important to note that we also observed greater rigidity in participants experiencing high levels of perceived chronic stress. Specifically, in Study 1 individuals with high levels of perceived chronic stress showed the same levels of perseveration regardless of the stress context. Though there are notable differences between our studies (e.g., different stressors), the underlying message is that chronic stress may be a key moderator in the relationship between acute stress and cognition.

In addition to behavioral data, we took repeated measurements of sAA in Study 2 to assess differences in women’s physiological acute stress response. We observed significant differences in the changes in sAA across time between the two conditions, as evidenced by a significant time by condition interaction. On average, women in the control condition showed a decrease in sAA following acute stress exposure, while those in the acute stress condition showed an increase across the same time period. In sum, our acute stress manipulation resulted in different patterns of change in sAA between conditions.

Further, we identified sAA responders and sAA non-responders. This allowed us to show a significant negative correlation between perceived chronic stress levels and perseverative errors for these women who were sAA responders. In effect, our data suggested that high perceived chronic stress was associated with fewer perseverative errors for sAA responders. Tanaka et al.^47^ suggest that the magnitude of the sAA response can be an indicator of an adaptive stress response, with change in sAA in responders indicating the preservation of normative hormonal responsiveness to stress. This might explain why sAA responders who were experiencing high levels of perceived chronic stress showed reduced perseveration. Unlike Liston et al., we did not experimentally manipulate chronic stress, and our sample of sAA responders is very small (n = 7), so no claims can be made from our data. Additionally, Study 2 was underpowered. Thus, these results must be approached with caution and replicated with a larger sample before any firm conclusions are drawn. However, future work should interrogate the role of sAA response in stress effects related to cognitive flexibility.

Overall, our work is consistent with prior observations that PFC function is impacted by both acute and chronic stress, with implications for set-shifting performance^3,19^. Since the dlPFC is implicated in flexible behavior following both acute^3^ and chronic stress exposure^19^, future research should elucidate the role of the dlPFC in the interaction of acute and chronic stress. Our findings provide support for the proposal outlined by McEwen^58^, that the acute stress response is adaptive but chronic stress disrupts the acute stress response.

### Associations between perceived chronic stress and subjective stress following manipulation

In Study 1, perceived chronic stress was positively correlated with subjective stress levels pre- and post-manipulation, but in Study 2, subjective stress, difficulty, and discomfort were uncorrelated with ratings of perceived chronic stress. This may be due to differences in the timing of the stress measures. In Study 2, perceived chronic stress was measured approximately 2 weeks prior to the acute stress induction and subjective stress assessments. In contrast, perceived chronic stress and subjective stress were measured on the same day in Study 1, which may account for some of the shared variance between the two measures. In sum, subjective appraisals following acute stress exposure appeared to be correlated with individuals’ current, but not previous, levels of perceived chronic stress.

Prior research by Stawski et al.^25^ has shown that perceived chronic stress is associated with greater reactivity to stressful life experiences. However, Stawski et al. looked at only experiences within 10 days of measuring perceived chronic stress. It is possible that the 2-week (on average) gap between our perceived chronic stress and subjective stress measurement may have influenced our findings. Future research should explore longitudinal relationships between perceived chronic stress and subjective stress reactivity.

### Perseveration under acute stress occurs in both male and female participants

Sex differences have emerged in previous work on the effects of acute stress on perseveration^3,4^. In particular, both Kalia et al. and Shields et al. reported that acute stress increased perseveration in male, but not female, participants. Our data in Study 1 diverge from this pattern; no sex differences in acute stress effects emerged. Some prior reports have also not observed sex differences in acute stress effects on cognitive flexibility^59,60^. Yet, our data make it possible to speculate that sex or gender differences in perceived chronic stress may play a role in this divergence in cognitive acute stress effects. Female participants in Study 1 had higher perceived chronic stress scores (*M* = 16.65; *SD* = 7.26) than male participants (*M* = 13.80; *SD* = 5.85). Furthermore, women and those assigned female at birth report elevated stress-related mental and physical health problems^61^ and greater stress across their lifespan^62^. Thus, sex and gender-associated differences in the effects of stress warrant further investigation.

### Limitations and future directions

Our work must be considered in light of its limitations. First, we did not obtain measures of cortisol during acute stress induction in either study. Thus, we cannot make any claims about the role of the HPA axis in the effects observed here. Second, we used a self-report measure to assess chronic stress, though the PSS has been extensively used and validated^20,63^ and has been found to be associated with changes in dlPFC function and set-shifting^19^. In light of this, we limit the scope of our conclusions to the individual’s perception of chronic stress, rather than chronic stress as measured by a life event schedule or physiology. Future work should measure hair cortisol to disentangle the psychological and physiological mechanisms that might underlie the interaction seen here. Third, Study 2 was underpowered. Therefore, the results need to be interpreted with extreme caution and replication of the findings in a larger study is essential. Fourth, our studies were conducted using undergraduate student populations, and it is unclear if these results will generalize to the broader population who may experience and appraise stress differently. Future work should investigate the interactive effects of acute and chronic stress on cognitive flexibility in larger and more diverse community. Fifth, previous literature states that race, ethnicity, gender, and sexual orientation have significant influence on physical and mental health due to the stress of stigma and discrimination^64–66^. Participants were predominantly white and no questions were asked regarding sexual orientation, so it is possible that these findings will not be generalizable. Future work should examine the differences in stressors among those with marginalized identities. Finally, we did not assess participants' mental health (e.g., depression or anxiety), so the findings may not extend to clinical populations. Future work should look at the interactive effects of acute and chronic stress within adult samples and diverse community samples to better understand the generalizability of the effects observed here.

### Summary of findings and conclusion

Nevertheless, the present studies extend the literature in three key ways. First, we provide evidence that the effect of acute stress on cognitive flexibility varies as a function of individuals’ perceived chronic stress. As researchers begin to consider moderating factors of the effects of acute stress on cognition^67^, our work highlights perceived chronic stress as an important variable of interest. Second, we extend work investigating the role of the sympathetic nervous system in acute stress effects on cognition^6,39,40^ by providing preliminary evidence for a connection between perceived chronic stress and cognitive flexibility in sAA responders. Finally, we found evidence for the interactive effects of stress on cognitive flexibility in both male and female participants within Study 1 and in women within Study 2, deepening our understanding of the role of sex and gender in cognitive stress effects. Taken together, the present studies suggest that it is important to consider perceived chronic stress within work on stress and cognitive flexibility. Perceived chronic stress may be particularly important for isolating the effects of acute stress on cognitive flexibility in women, as they report elevated chronic stress levels^62^ and increased mental health problems linked to stress and cognitive inflexibility^61,68^.

## Data Availability

The datasets analyzed during the current studies are available from the corresponding author on reasonable request.
